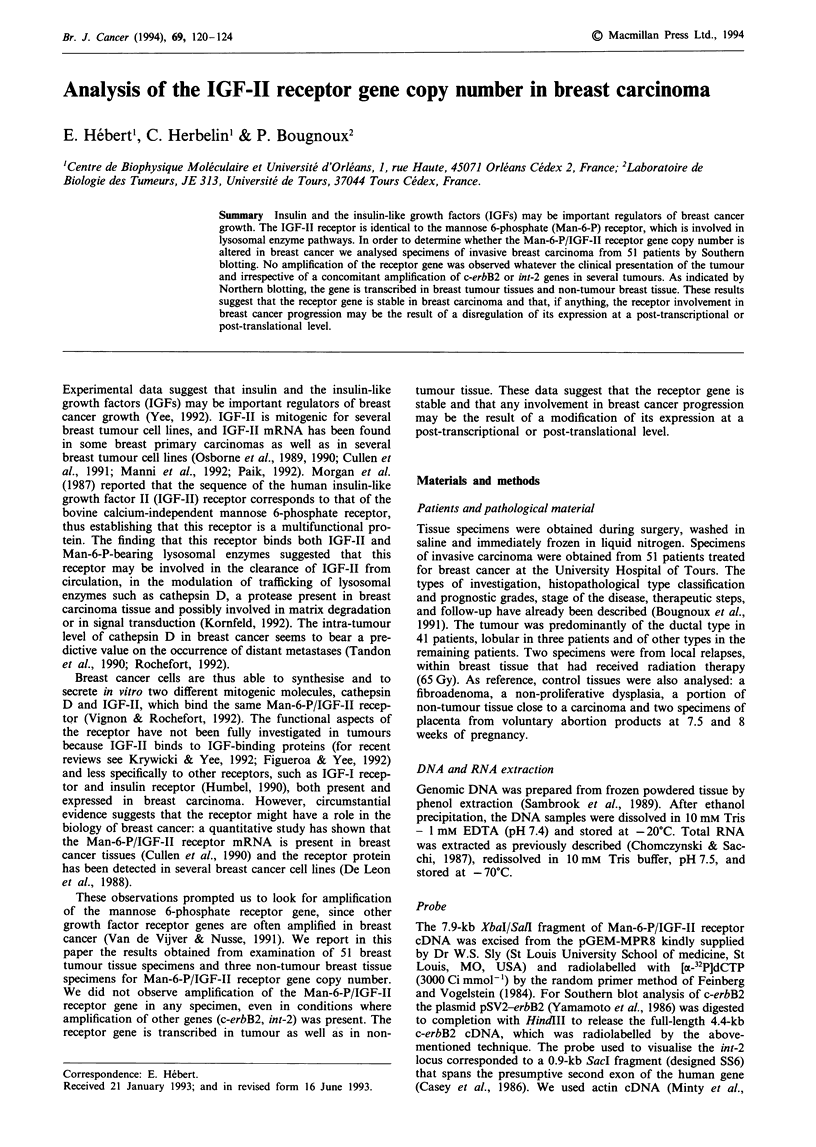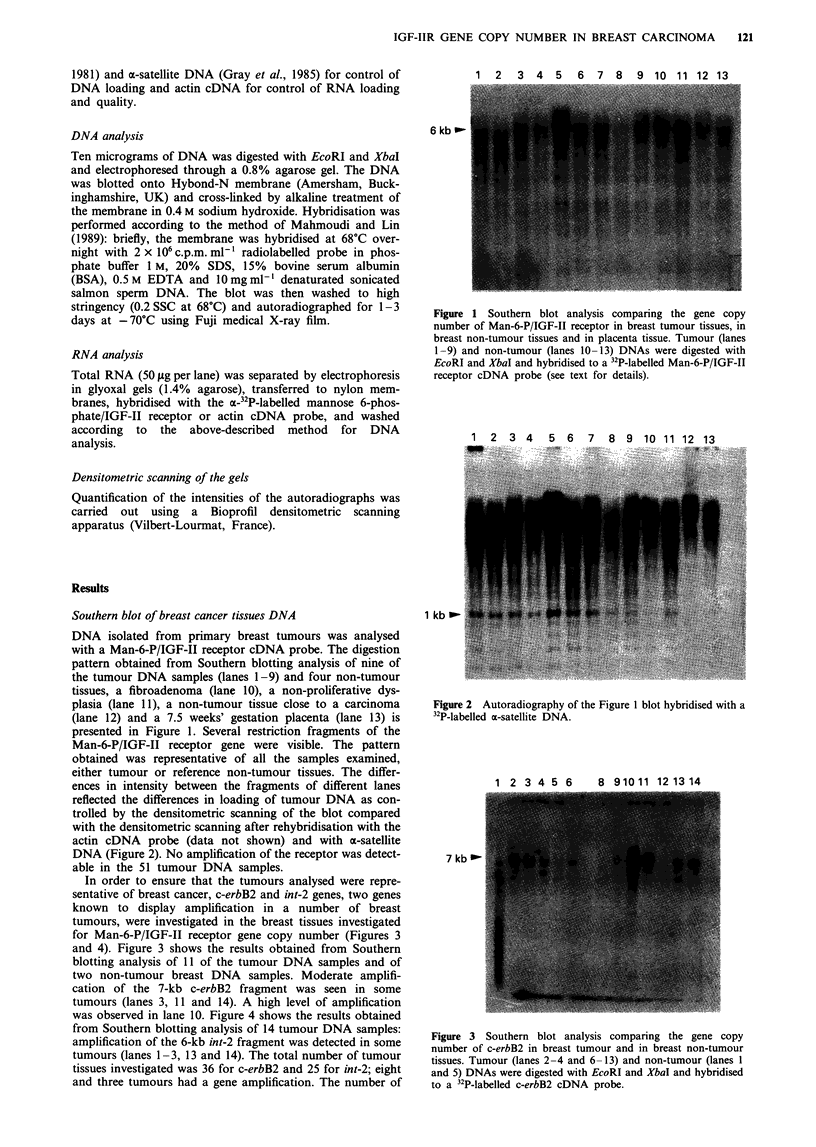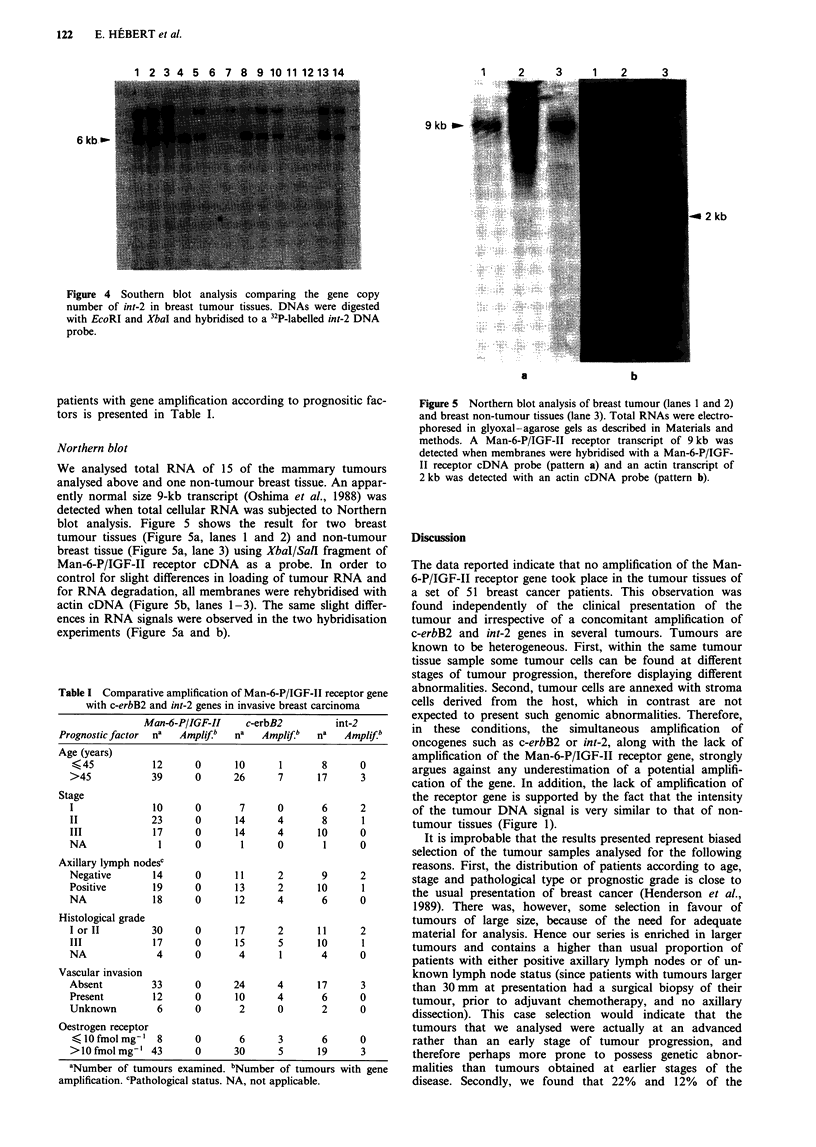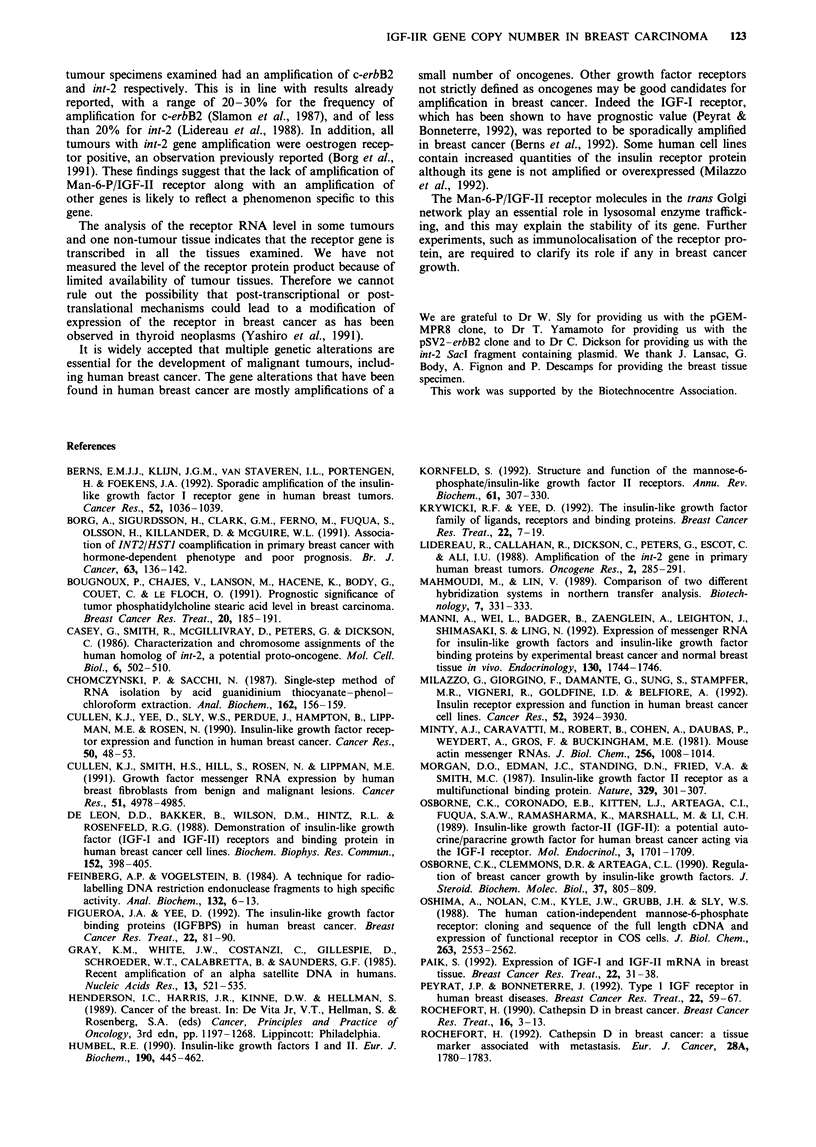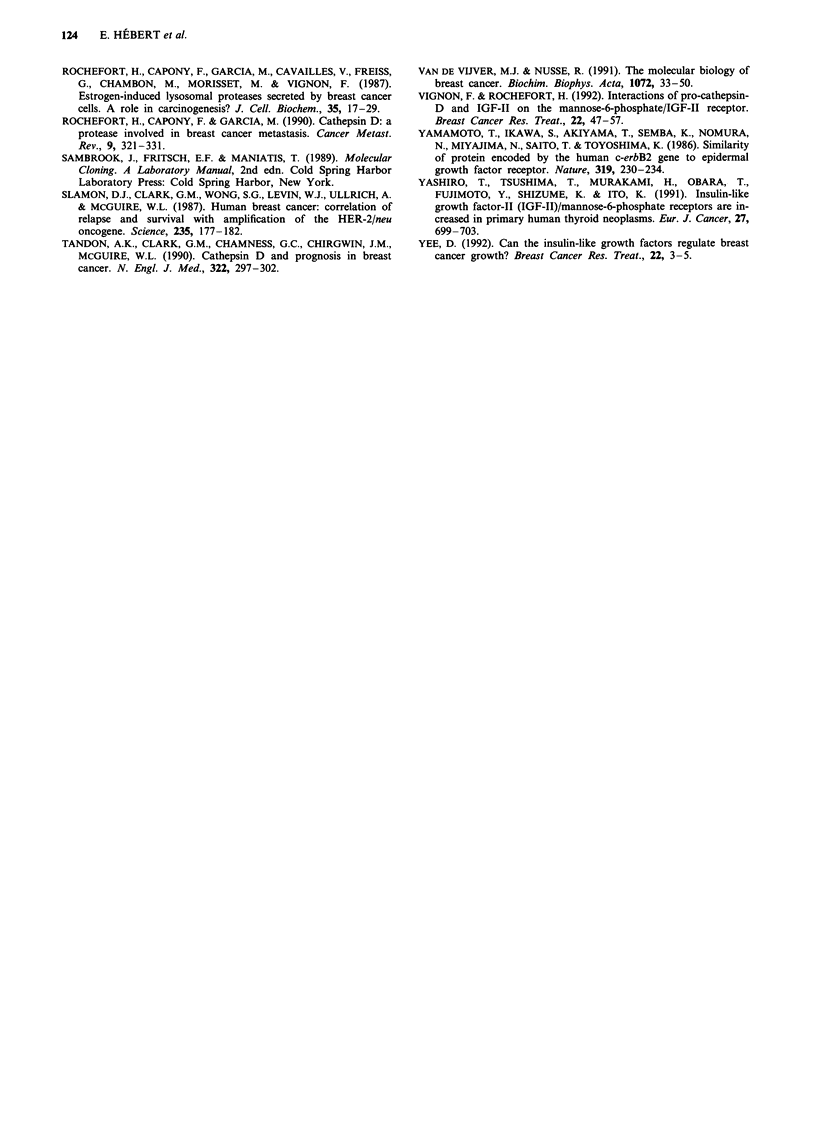# Analysis of the IGF-II receptor gene copy number in breast carcinoma.

**DOI:** 10.1038/bjc.1994.19

**Published:** 1994-01

**Authors:** E. Hébert, C. Herbelin, P. Bougnoux

**Affiliations:** Centre de Biophysique Moléculaire et Université d'Orléans, France.

## Abstract

**Images:**


Br. J. Cancer (1994), 69, 120–124 © Macmillan Press Ltd., 1994

### Analysis of the IGF-II receptor gene copy number in breast carcinoma

E. Hébert[1], C. Herbelin[1] & P. Bougnoux[2]

[1]*Centre de Biophysique Moléculaire et Université d'Orléans, 1, rue Haute, 45071 Orléans Cédex 2, France;* [2]*Laboratoire de Biologie des Tumeurs, JE 313, Université de Tours, 37044 Tours Cédex, France.*

**Summary** Insulin and the insulin-like growth factors (IGFs) may be important regulators of breast cancer growth. The IGF-II receptor is identical to the mannose 6-phosphate (Man-6-P) receptor, which is involved in lysosomal enzyme pathways. In order to determine whether the Man-6-P/IGF-II receptor gene copy number is altered in breast cancer we analysed specimens of invasive breast carcinoma from 51 patients by Southern blotting. No amplification of the receptor gene was observed whatever the clinical presentation of the tumour and irrespective of a concomitant amplification of c-*erb*B2 or *int*-2 genes in several tumours. As indicated by Northern blotting, the gene is transcribed in breast tumour tissues and non-tumour breast tissue. These results suggest that the receptor gene is stable in breast carcinoma and that, if anything, the receptor involvement in breast cancer progression may be the result of a disregulation of its expression at a post-transcriptional or post-translational level.

Experimental data suggest that insulin and the insulin-like growth factors (IGFs) may be important regulators of breast cancer growth (Yee, 1992). IGF-II is mitogenic for several breast tumour cell lines, and IGF-II mRNA has been found in some breast primary carcinomas as well as in several breast tumour cell lines (Osborne *et al.*, 1989, 1990; Cullen *et al.*, 1991; Manni *et al.*, 1992; Paik, 1992). Morgan *et al.* (1987) reported that the sequence of the human insulin-like growth factor II (IGF-II) receptor corresponds to that of the bovine calcium-independent mannose 6-phosphate receptor, thus establishing that this receptor is a multifunctional protein. The finding that this receptor binds both IGF-II and Man-6-P-bearing lysosomal enzymes suggested that this receptor may be involved in the clearance of IGF-II from circulation, in the modulation of trafficking of lysosomal enzymes such as cathepsin D, a protease present in breast carcinoma tissue and possibly involved in matrix degradation or in signal transduction (Kornfeld, 1992). The intra-tumour level of cathepsin D in breast cancer seems to bear a predictive value on the occurrence of distant metastases (Tandon *et al.*, 1990; Rochefort, 1992).

Breast cancer cells are thus able to synthesise and to secrete *in vitro* two different mitogenic molecules, cathepsin D and IGF-II, which bind the same Man-6-P/IGF-II receptor (Vignon & Rochefort, 1992). The functional aspects of the receptor have not been fully investigated in tumours because IGF-II binds to IGF-binding proteins (for recent reviews see Krywicki & Yee, 1992; Figueroa & Yee, 1992) and less specifically to other receptors, such as IGF-I receptor and insulin receptor (Humbel, 1990), both present and expressed in breast carcinoma. However, circumstantial evidence suggests that the receptor might have a role in the biology of breast cancer: a quantitative study has shown that the Man-6-P/IGF-II receptor mRNA is present in breast cancer tissues (Cullen *et al.*, 1990) and the receptor protein has been detected in several breast cancer cell lines (De Leon *et al.*, 1988).

These observations prompted us to look for amplification of the mannose 6-phosphate receptor gene, since other growth factor receptor genes are often amplified in breast cancer (Van de Vijver & Nusse, 1991). We report in this paper the results obtained from examination of 51 breast tumour tissue specimens and three non-tumour breast tissue specimens for Man-6-P/IGF-II receptor gene copy number. We did not observe amplification of the Man-6-P/IGF-II receptor gene in any specimen, even in conditions where amplification of other genes (c-*erb*B2, *int*-2) was present. The receptor gene is transcribed in tumour as well as in non-tumour tissue. These data suggest that the receptor gene is stable and that any involvement in breast cancer progression may be the result of a modification of its expression at a post-transcriptional or post-translational level.

### Materials and methods

### *Patients and pathological material*

Tissue specimens were obtained during surgery, washed in saline and immediately frozen in liquid nitrogen. Specimens of invasive carcinoma were obtained from 51 patients treated for breast cancer at the University Hospital of Tours. The types of investigation, histopathological type classification and prognostic grades, stage of the disease, therapeutic steps, and follow-up have already been described (Bougnoux *et al.*, 1991). The tumour was predominantly of the ductal type in 41 patients, lobular in three patients and of other types in the remaining patients. Two specimens were from local relapses, within breast tissue that had received radiation therapy (65 Gy). As reference, control tissues were also analysed: a fibroadenoma, a non-proliferative dysplasia, a portion of non-tumour tissue close to a carcinoma and two specimens of placenta from voluntary abortion products at 7.5 and 8 weeks of pregnancy.

### *DNA and RNA extraction*

Genomic DNA was prepared from frozen powdered tissue by phenol extraction (Sambrook *et al.*, 1989). After ethanol precipitation, the DNA samples were dissolved in 10 mM Tris – 1 mM EDTA (pH 7.4) and stored at −20°C. Total RNA was extracted as previously described (Chomczynski & Sacchi, 1987), redissolved in 10 mM Tris buffer, pH 7.5, and stored at −70°C.

### *Probe*

The 7.9-kb *XbaI*/*SalI* fragment of Man-6-P/IGF-II receptor cDNA was excised from the pGEM-MPR8 kindly supplied by Dr W.S. Sly (St Louis University School of medicine, St Louis, MO, USA) and radiolabelled with [$\alpha$-$^{32}$P]dCTP (3000 Ci mmol$^{-1}$) by the random primer method of Feinberg and Vogelstein (1984). For Southern blot analysis of c-*erb*B2 the plasmid pSV2-*erb*B2 (Yamamoto *et al.*, 1986) was digested to completion with *Hind*III to release the full-length 4.4-kb c-*erb*B2 cDNA, which was radiolabelled by the above-mentioned technique. The probe used to visualise the *int*-2 locus corresponded to a 0.9-kb *SacI* fragment (designed SS6) that spans the presumptive second exon of the human gene (Casey *et al.*, 1986). We used actin cDNA (Minty *et al.*,

Correspondence: E. Hébert.
Received 21 January 1993; and in revised form 16 June 1993.

1981) and α-satellite DNA (Gray *et al.*, 1985) for control of DNA loading and actin cDNA for control of RNA loading and quality.

*DNA analysis*

Ten micrograms of DNA was digested with *Eco*RI and *Xba*I and electrophoresed through a 0.8% agarose gel. The DNA was blotted onto Hybond-N membrane (Amersham, Buckinghamshire, UK) and cross-linked by alkaline treatment of the membrane in 0.4 M sodium hydroxide. Hybridisation was performed according to the method of Mahmoudi and Lin (1989): briefly, the membrane was hybridised at 68°C overnight with $2 \times 10^6$ c.p.m. $ml^{-1}$ radiolabelled probe in phosphate buffer 1 M, 20% SDS, 15% bovine serum albumin (BSA), 0.5 M EDTA and 10 mg $ml^{-1}$ denaturated sonicated salmon sperm DNA. The blot was then washed to high stringency (0.2 SSC at 68°C) and autoradiographed for 1–3 days at −70°C using Fuji medical X-ray film.

*RNA analysis*

Total RNA (50 μg per lane) was separated by electrophoresis in glyoxal gels (1.4% agarose), transferred to nylon membranes, hybridised with the α-$^{32}$P-labelled mannose 6-phosphate/IGF-II receptor or actin cDNA probe, and washed according to the above-described method for DNA analysis.

*Densitometric scanning of the gels*

Quantification of the intensities of the autoradiographs was carried out using a Bioprofil densitometric scanning apparatus (Vilbert-Lourmat, France).

### Results

*Southern blot of breast cancer tissues DNA*

DNA isolated from primary breast tumours was analysed with a Man-6-P/IGF-II receptor cDNA probe. The digestion pattern obtained from Southern blotting analysis of nine of the tumour DNA samples (lanes 1–9) and four non-tumour tissues, a fibroadenoma (lane 10), a non-proliferative dysplasia (lane 11), a non-tumour tissue close to a carcinoma (lane 12) and a 7.5 weeks' gestation placenta (lane 13) is presented in Figure 1. Several restriction fragments of the Man-6-P/IGF-II receptor gene were visible. The pattern obtained was representative of all the samples examined, either tumour or reference non-tumour tissues. The differences in intensity between the fragments of different lanes reflected the differences in loading of tumour DNA as controlled by the densitometric scanning of the blot compared with the densitometric scanning after rehybridisation with the actin cDNA probe (data not shown) and with α-satellite DNA (Figure 2). No amplification of the receptor was detectable in the 51 tumour DNA samples.

In order to ensure that the tumours analysed were representative of breast cancer, c-*erb*B2 and *int*-2 genes, two genes known to display amplification in a number of breast tumours, were investigated in the breast tissues investigated for Man-6-P/IGF-II receptor gene copy number (Figures 3 and 4). Figure 3 shows the results obtained from Southern blotting analysis of 11 of the tumour DNA samples and of two non-tumour breast DNA samples. Moderate amplification of the 7-kb c-*erb*B2 fragment was seen in some tumours (lanes 3, 11 and 14). A high level of amplification was observed in lane 10. Figure 4 shows the results obtained from Southern blotting analysis of 14 tumour DNA samples: amplification of the 6-kb *int*-2 fragment was detected in some tumours (lanes 1–3, 13 and 14). The total number of tumour tissues investigated was 36 for c-*erb*B2 and 25 for *int*-2; eight and three tumours had a gene amplification. The number of

**Figure 1** Southern blot analysis comparing the gene copy number of Man-6-P/IGF-II receptor in breast tumour tissues, in breast non-tumour tissues and in placenta tissue. Tumour (lanes 1–9) and non-tumour (lanes 10–13) DNAs were digested with *Eco*RI and *Xba*I and hybridised to a $^{32}$P-labelled Man-6-P/IGF-II receptor cDNA probe (see text for details).

**Figure 2** Autoradiography of the Figure 1 blot hybridised with a $^{32}$P-labelled α-satellite DNA.

**Figure 3** Southern blot analysis comparing the gene copy number of c-*erb*B2 in breast tumour and in breast non-tumour tissues. Tumour (lanes 2–4 and 6–13) and non-tumour (lanes 1 and 5) DNAs were digested with *Eco*RI and *Xba*I and hybridised to a $^{32}$P-labelled c-*erb*B2 cDNA probe.

**Figure 4** Southern blot analysis comparing the gene copy number of *int*-2 in breast tumour tissues. DNAs were digested with *Eco*RI and *Xba*I and hybridised to a $^{32}$P-labelled *int*-2 DNA probe.

**Figure 5** Northern blot analysis of breast tumour (lanes 1 and 2) and breast non-tumour tissues (lane 3). Total RNAs were electrophoresed in glyoxal–agarose gels as described in Materials and methods. A Man-6-P/IGF-II receptor transcript of 9 kb was detected when membranes were hybridised with a Man-6-P/IGF-II receptor cDNA probe (pattern **a**) and an actin transcript of 2 kb was detected with an actin cDNA probe (pattern **b**).

patients with gene amplification according to prognositic factors is presented in Table I.

*Northern blot*

We analysed total RNA of 15 of the mammary tumours analysed above and one non-tumour breast tissue. An apparently normal size 9-kb transcript (Oshima *et al.*, 1988) was detected when total cellular RNA was subjected to Northern blot analysis. Figure 5 shows the result for two breast tumour tissues (Figure 5a, lanes 1 and 2) and non-tumour breast tissue (Figure 5a, lane 3) using *Xba*I/*Sal*I fragment of Man-6-P/IGF-II receptor cDNA as a probe. In order to control for slight differences in loading of tumour RNA and for RNA degradation, all membranes were rehybridised with actin cDNA (Figure 5b, lanes 1–3). The same slight differences in RNA signals were observed in the two hybridisation experiments (Figure 5a and b).

**Table I** Comparative amplification of Man-6-P/IGF-II receptor gene with c-*erb*B2 and *int*-2 genes in invasive breast carcinoma

| Prognostic factor | *Man-6-P/IGF-II* n[a] | *Man-6-P/IGF-II* *Amplif.*[b] | c-erb*B2* n[a] | c-erb*B2* *Amplif.*[b] | int-*2* n[a] | int-*2* *Amplif.*[b] |
|---|---|---|---|---|---|---|
| Age (years) | | | | | | |
| ⩽45 | 12 | 0 | 10 | 1 | 8 | 0 |
| >45 | 39 | 0 | 26 | 7 | 17 | 3 |
| Stage | | | | | | |
| I | 10 | 0 | 7 | 0 | 6 | 2 |
| II | 23 | 0 | 14 | 4 | 8 | 1 |
| III | 17 | 0 | 14 | 4 | 10 | 0 |
| NA | 1 | 0 | 1 | 0 | 1 | 0 |
| Axillary lymph nodes[c] | | | | | | |
| Negative | 14 | 0 | 11 | 2 | 9 | 2 |
| Positive | 19 | 0 | 13 | 2 | 10 | 1 |
| NA | 18 | 0 | 12 | 4 | 6 | 0 |
| Histological grade | | | | | | |
| I or II | 30 | 0 | 17 | 2 | 11 | 2 |
| III | 17 | 0 | 15 | 5 | 10 | 1 |
| NA | 4 | 0 | 4 | 1 | 4 | 0 |
| Vascular invasion | | | | | | |
| Absent | 33 | 0 | 24 | 4 | 17 | 3 |
| Present | 12 | 0 | 10 | 4 | 6 | 0 |
| Unknown | 6 | 0 | 2 | 0 | 2 | 0 |
| Oestrogen receptor | | | | | | |
| ⩽10 fmol mg$^{-1}$ | 8 | 0 | 6 | 3 | 6 | 0 |
| >10 fmol mg$^{-1}$ | 43 | 0 | 30 | 5 | 19 | 3 |

[a]Number of tumours examined. [b]Number of tumours with gene amplification. [c]Pathological status. NA, not applicable.

### Discussion

The data reported indicate that no amplification of the Man-6-P/IGF-II receptor gene took place in the tumour tissues of a set of 51 breast cancer patients. This observation was found independently of the clinical presentation of the tumour and irrespective of a concomitant amplification of c-*erb*B2 and *int*-2 genes in several tumours. Tumours are known to be heterogeneous. First, within the same tumour tissue sample some tumour cells can be found at different stages of tumour progression, therefore displaying different abnormalities. Second, tumour cells are annexed with stroma cells derived from the host, which in contrast are not expected to present such genomic abnormalities. Therefore, in these conditions, the simultaneous amplification of oncogenes such as c-*erb*B2 or *int*-2, along with the lack of amplification of the Man-6-P/IGF-II receptor gene, strongly argues against any underestimation of a potential amplification of the gene. In addition, the lack of amplification of the receptor gene is supported by the fact that the intensity of the tumour DNA signal is very similar to that of non-tumour tissues (Figure 1).

It is improbable that the results presented represent biased selection of the tumour samples analysed for the following reasons. First, the distribution of patients according to age, stage and pathological type or prognostic grade is close to the usual presentation of breast cancer (Henderson *et al.*, 1989). There was, however, some selection in favour of tumours of large size, because of the need for adequate material for analysis. Hence our series is enriched in larger tumours and contains a higher than usual proportion of patients with either positive axillary lymph nodes or of unknown lymph node status (since patients with tumours larger than 30 mm at presentation had a surgical biopsy of their tumour, prior to adjuvant chemotherapy, and no axillary dissection). This case selection would indicate that the tumours that we analysed were actually at an advanced rather than an early stage of tumour progression, and therefore perhaps more prone to possess genetic abnormalities than tumours obtained at earlier stages of the disease. Secondly, we found that 22% and 12% of the tumour specimens examined had an amplification of c-*erb*B2 and *int*-2 respectively. This is in line with results already reported, with a range of 20–30% for the frequency of amplification for c-*erb*B2 (Slamon *et al.*, 1987), and of less than 20% for *int*-2 (Lidereau *et al.*, 1988). In addition, all tumours with *int*-2 gene amplification were oestrogen receptor positive, an observation previously reported (Borg *et al.*, 1991). These findings suggest that the lack of amplification of Man-6-P/IGF-II receptor along with an amplification of other genes is likely to reflect a phenomenon specific to this gene.

The analysis of the receptor RNA level in some tumours and one non-tumour tissue indicates that the receptor gene is transcribed in all the tissues examined. We have not measured the level of the receptor protein product because of limited availability of tumour tissues. Therefore we cannot rule out the possibility that post-transcriptional or post-translational mechanisms could lead to a modification of expression of the receptor in breast cancer as has been observed in thyroid neoplasms (Yashiro *et al.*, 1991).

It is widely accepted that multiple genetic alterations are essential for the development of malignant tumours, including human breast cancer. The gene alterations that have been found in human breast cancer are mostly amplifications of a small number of oncogenes. Other growth factor receptors not strictly defined as oncogenes may be good candidates for amplification in breast cancer. Indeed the IGF-I receptor, which has been shown to have prognostic value (Peyrat & Bonneterre, 1992), was reported to be sporadically amplified in breast cancer (Berns *et al.*, 1992). Some human cell lines contain increased quantities of the insulin receptor protein although its gene is not amplified or overexpressed (Milazzo *et al.*, 1992).

The Man-6-P/IGF-II receptor molecules in the *trans* Golgi network play an essential role in lysosomal enzyme trafficking, and this may explain the stability of its gene. Further experiments, such as immunolocalisation of the receptor protein, are required to clarify its role if any in breast cancer growth.

We are grateful to Dr W. Sly for providing us with the pGEM-MPR8 clone, to Dr T. Yamamoto for providing us with the pSV2-*erb*B2 clone and to Dr C. Dickson for providing us with the *int*-2 *Sac*I fragment containing plasmid. We thank J. Lansac, G. Body, A. Fignon and P. Descamps for providing the breast tissue specimen.

This work was supported by the Biotechnocentre Association.

### References

BERNS, E.M.J.J., KLIJN, J.G.M., VAN STAVEREN, I.L., PORTENGEN, H. & FOEKENS, J.A. (1992). Sporadic amplification of the insulin-like growth factor I receptor gene in human breast tumors. *Cancer Res.*, **52**, 1036–1039.

BORG, A., SIGURDSSON, H., CLARK, G.M., FERNO, M., FUQUA, S., OLSSON, H., KILLANDER, D. & MCGUIRE, W.L. (1991). Association of *INT2/HST1* coamplification in primary breast cancer with hormone-dependent phenotype and poor prognosis. *Br. J. Cancer*, **63**, 136–142.

BOUGNOUX, P., CHAJES, V., LANSON, M., HACENE, K., BODY, G., COUET, C. & LE FLOCH, O. (1991). Prognostic significance of tumor phosphatidylcholine stearic acid level in breast carcinoma. *Breast Cancer Res. Treat.*, **20**, 185–191.

CASEY, G., SMITH, R., MCGILLIVRAY, D., PETERS, G. & DICKSON, C. (1986). Characterization and chromosome assignments of the human homolog of *int*-2, a potential proto-oncogene. *Mol. Cell. Biol.*, **6**, 502–510.

CHOMCZYNSKI, P. & SACCHI, N. (1987). Single-step method of RNA isolation by acid guanidinium thiocyanate–phenol–chloroform extraction. *Anal. Biochem.*, **162**, 156–159.

CULLEN, K.J., YEE, D., SLY, W.S., PERDUE, J., HAMPTON, B., LIPPMAN, M.E. & ROSEN, N. (1990). Insulin-like growth factor receptor expression and function in human breast cancer. *Cancer Res.*, **50**, 48–53.

CULLEN, K.J., SMITH, H.S., HILL, S., ROSEN, N. & LIPPMAN, M.E. (1991). Growth factor messenger RNA expression by human breast fibroblasts from benign and malignant lesions. *Cancer Res.*, **51**, 4978–4985.

DE LEON, D.D., BAKKER, B., WILSON, D.M., HINTZ, R.L. & ROSENFELD, R.G. (1988). Demonstration of insulin-like growth factor (IGF-I and IGF-II) receptors and binding protein in human breast cancer cell lines. *Biochem. Biophys. Res. Commun.*, **152**, 398–405.

FEINBERG, A.P. & VOGELSTEIN, B. (1984). A technique for radiolabelling DNA restriction endonuclease fragments to high specific activity. *Anal. Biochem.*, **132**, 6–13.

FIGUEROA, J.A. & YEE, D. (1992). The insulin-like growth factor binding proteins (IGFBPS) in human breast cancer. *Breast Cancer Res. Treat.*, **22**, 81–90.

GRAY, K.M., WHITE, J.W., COSTANZI, C., GILLESPIE, D., SCHROEDER, W.T., CALABRETTA, B. & SAUNDERS, G.F. (1985). Recent amplification of an alpha satellite DNA in humans. *Nucleic Acids Res.*, **13**, 521–535.

HENDERSON, I.C., HARRIS, J.R., KINNE, D.W. & HELLMAN, S. (1989). Cancer of the breast. In: De Vita Jr, V.T., Hellman, S. & Rosenberg, S.A. (eds) *Cancer, Principles and Practice of Oncology*, 3rd edn, pp. 1197–1268. Lippincott: Philadelphia.

HUMBEL, R.E. (1990). Insulin-like growth factors I and II. *Eur. J. Biochem.*, **190**, 445–462.

KORNFELD, S. (1992). Structure and function of the mannose-6-phosphate/insulin-like growth factor II receptors. *Annu. Rev. Biochem.*, **61**, 307–330.

KRYWICKI, R.F. & YEE, D. (1992). The insulin-like growth factor family of ligands, receptors and binding proteins. *Breast Cancer Res. Treat.*, **22**, 7–19.

LIDEREAU, R., CALLAHAN, R., DICKSON, C., PETERS, G., ESCOT, C. & ALI, I.U. (1988). Amplification of the *int*-2 gene in primary human breast tumors. *Oncogene Res.*, **2**, 285–291.

MAHMOUDI, M., & LIN, V. (1989). Comparison of two different hybridization systems in northern transfer analysis. *Biotechnology*, **7**, 331–333.

MANNI, A., WEI, L., BADGER, B., ZAENGLEIN, A., LEIGHTON, J., SHIMASAKI, S. & LING, N. (1992). Expression of messenger RNA for insulin-like growth factors and insulin-like growth factor binding proteins by experimental breast cancer and normal breast tissue *in vivo*. *Endocrinology*, **130**, 1744–1746.

MILAZZO, G., GIORGINO, F., DAMANTE, G., SUNG, S., STAMPFER, M.R., VIGNERI, R., GOLDFINE, I.D. & BELFIORE, A. (1992). Insulin receptor expression and function in human breast cancer cell lines. *Cancer Res.*, **52**, 3924–3930.

MINTY, A.J., CARAVATTI, M., ROBERT, B., COHEN, A., DAUBAS, P., WEYDERT, A., GROS, F. & BUCKINGHAM, M.E. (1981). Mouse actin messenger RNAs. *J. Biol. Chem.*, **256**, 1008–1014.

MORGAN, D.O., EDMAN, J.C., STANDING, D.N., FRIED, V.A. & SMITH, M.C. (1987). Insulin-like growth factor II receptor as a multifunctional binding protein. *Nature*, **329**, 301–307.

OSBORNE, C.K., CORONADO, E.B., KITTEN, L.J., ARTEAGA, C.I., FUQUA, S.A.W., RAMASHARMA, K., MARSHALL, M. & LI, C.H. (1989). Insulin-like growth factor-II (IGF-II): a potential autocrine/paracrine growth factor for human breast cancer acting via the IGF-I receptor. *Mol. Endocrinol.*, **3**, 1701–1709.

OSBORNE, C.K., CLEMMONS, D.R. & ARTEAGA, C.L. (1990). Regulation of breast cancer growth by insulin-like growth factors. *J. Steroid. Biochem. Molec. Biol.*, **37**, 805–809.

OSHIMA, A., NOLAN, C.M., KYLE, J.W., GRUBB, J.H. & SLY, W.S. (1988). The human cation-independent mannose-6-phosphate receptor: cloning and sequence of the full length cDNA and expression of functional receptor in COS cells. *J. Biol. Chem.*, **263**, 2553–2562.

PAIK, S. (1992). Expression of IGF-I and IGF-II mRNA in breast tissue. *Breast Cancer Res. Treat.*, **22**, 31–38.

PEYRAT, J.P. & BONNETERRE, J. (1992). Type 1 IGF receptor in human breast diseases. *Breast Cancer Res. Treat.*, **22**, 59–67.

ROCHEFORT, H. (1990). Cathepsin D in breast cancer. *Breast Cancer Res. Treat.*, **16**, 3–13.

ROCHEFORT, H. (1992). Cathepsin D in breast cancer: a tissue marker associated with metastasis. *Eur. J. Cancer*, **28A**, 1780–1783.

ROCHEFORT, H., CAPONY, F., GARCIA, M., CAVAILLES, V., FREISS, G., CHAMBON, M., MORISSET, M. & VIGNON, F. (1987). Estrogen-induced lysosomal proteases secreted by breast cancer cells. A role in carcinogenesis? *J. Cell. Biochem.*, **35**, 17–29.

ROCHEFORT, H., CAPONY, F. & GARCIA, M. (1990). Cathepsin D: a protease involved in breast cancer metastasis. *Cancer Metast. Rev.*, **9**, 321–331.

SAMBROOK, J., FRITSCH, E.F. & MANIATIS, T. (1989). *Molecular Cloning. A Laboratory Manual*, 2nd edn. Cold Spring Harbor Laboratory Press: Cold Spring Harbor, New York.

SLAMON, D.J., CLARK, G.M., WONG, S.G., LEVIN, W.J., ULLRICH, A. & MCGUIRE, W.L. (1987). Human breast cancer: correlation of relapse and survival with amplification of the HER-2/*neu* oncogene. *Science*, **235**, 177–182.

TANDON, A.K., CLARK, G.M., CHAMNESS, G.C., CHIRGWIN, J.M., MCGUIRE, W.L. (1990). Cathepsin D and prognosis in breast cancer. *N. Engl. J. Med.*, **322**, 297–302.

VAN DE VIJVER, M.J. & NUSSE, R. (1991). The molecular biology of breast cancer. *Biochim. Biophys. Acta*, **1072**, 33–50.

VIGNON, F. & ROCHEFORT, H. (1992). Interactions of pro-cathepsin-D and IGF-II on the mannose-6-phosphate/IGF-II receptor. *Breast Cancer Res. Treat.*, **22**, 47–57.

YAMAMOTO, T., IKAWA, S., AKIYAMA, T., SEMBA, K., NOMURA, N., MIYAJIMA, N., SAITO, T. & TOYOSHIMA, K. (1986). Similarity of protein encoded by the human c-*erb*B2 gene to epidermal growth factor receptor. *Nature*, **319**, 230–234.

YASHIRO, T., TSUSHIMA, T., MURAKAMI, H., OBARA, T., FUJIMOTO, Y., SHIZUME, K. & ITO, K. (1991). Insulin-like growth factor-II (IGF-II)/mannose-6-phosphate receptors are increased in primary human thyroid neoplasms. *Eur. J. Cancer*, **27**, 699–703.

YEE, D. (1992). Can the insulin-like growth factors regulate breast cancer growth? *Breast Cancer Res. Treat.*, **22**, 3–5.